# Cell Cycle-Dependent Expression Dynamics of G1/S Specific Cyclin, Cellulose Synthase and Cellulase in the Dinoflagellate *Prorocentrum donghaiense*

**DOI:** 10.3389/fmicb.2017.01118

**Published:** 2017-06-20

**Authors:** Xinguo Shi, Minglei Ma, Senjie Lin

**Affiliations:** ^1^State Key Laboratory of Marine Environmental Science, College of Ocean and Earth Sciences, Xiamen UniversityXiamen, China; ^2^Department of Marine Sciences, University of Connecticut, GrotonCT, United States

**Keywords:** cyclin, cellulase, cellulose synthesis, *Prorocentrum donghaiense*, gene regulation

## Abstract

Dinoflagellates undergo a typical eukaryotic cell cycle consisting of G1, S, G2, and M phases and some of the typical cell cycle related genes have been computationally identified. However, very few of these genes have been experimentally linked to the cell cycle phases. Besides, although thecate dinoflagellates are known to possess theca composed of cellulose, information on cellulose synthesis and degradation associated with the cell cycle is also limited. In this study, we isolated G1/S cyclin, cellulose synthase and cellulase encoding genes in dinoflagellate *Prorocentrum donghaiense*. Further, using reverse transcription quantitative PCR (RT-qPCR), we characterized the expression profiles of the three genes throughout the cell cycle. All three showed clear expression dynamics throughout the cell cycle, with fold changes of 26, 2.4 and 9.3 for G1/S cyclin, cellulose synthase and cellulase gene, respectively. The transcript abundance of G1/S cyclin increased in late G1 phase and dropped in early S phase, indicating that this protein is involved in the G1/S transition. Throughout the cell cycle, the average transcript level of cellulose synthase was 4.5-fold higher than that of cellulase. Cellulose synthase and cellulase gene expressions showed peak transcript abundances at middle G1 phase and G2M phase, respectively, indicating the respective roles of these enzymes in the growth of newly divided cells and in cytokinesis. Our results suggest that G1/S cyclin, cellulase, and cellulose synthase genes associated with G1/S transition, G2M, and G1 phases of the cell cycle and are candidates of biomarkers for assessing growth status of *P. donghaiense*.

## Introduction

Harmful algae blooms (HABs) are usually associated with high biomass accumulation of microalgae at the sea surface or in the water column (Anderson et al., 2012). To a large extent, algal biomass accumulation is consequence of rapid cell proliferation and population growth. Population growth of unicellular organisms such as bloom-forming phytoplankton results from active completion of the cell cycle, from G1 (first growth stage), S (DNA synthesis), G2 (second growth stage), and M (mitosis) followed by cytokinesis (cell division). To ensure that a cell divides only when the genetic material (DNA) has been completely duplicated and cellular condition is ready, the progression of the cell cycle stages is controlled by two major checkpoints, the G1/S checkpoint and the G2/M checkpoint (Johnson and Walker, 1999). These checkpoints are associated with the transitions from G1 to S and from G2 to M, respectively, and are regulated by specific cyclin-dependent kinases (CDKs). A CDK is activated by binding to its partner cyclin that triggers a cycle of phosphorylation and dephosphorylation at specific serine/threonine residues in the CDK (Serrano et al., 1993). As CDK abundances remain relatively constant throughout the cell cycle, the regulation of their activities hinges on the oscillation of the abundance of the cyclins (Arellano and Moreno, 1997). Thus, cyclins play a pivotal role in cell cycle regulation. Identifying the cell cycle phase-specific cyclins in HAB species would provide useful markers for monitoring the status of the bloom event toward understanding bloom formation mechanisms. The G1/S cyclin regulates the first transition of the cell cycle and is the key rate-limiting factor for cell proliferation (Edgar, 1994). However, the sequence and expression profile of this type cyclin has not been investigated in dinoflagellates, which are the dominant contributors of HABs in the global coastal ocean.

Another type of potential molecular markers are genes associated with cellulose synthesis and degradation in the case of thecate dinoflagellates that contain cellulosic amphiesmal vesicles underneath cell membrane (Lau et al., 2007). Conceivably, cellulose synthase and cellulase play an important role in cell wall formation and dissolution during cell growth and division, respectively. Cellulose synthase enzyme is a large protein in plasma membrane involved in the synthesis of cellulose (Jones et al., 2016). Cellulase, an enzyme responsible for cellulose degradation, is previously shown to be activated in G2/M transition in the dinoflagellate *C. cohnii* (Kwok and Wong, 2010). The high expression level of this enzyme is coupled with prolonged G2/M phase, suggesting that it is involved in the process of cytokinesis.

*Prorocentrum donghaiense* is a bloom-forming thecate dinoflagellate species. It forms massive blooms in the East China Sea coastal waters almost every year since 1998 (Lu et al., 2005). This species is genetically identical to *P. dentatum* based on nuclear rRNA genes and mitochondrial genes (Lin et al., 2006), which is widely distributed in Europe, America, Australia, and New Zealand (Guiry and Guiry, 2014). The blooms of *P. donghaiense* can extend 10,000 km^2^ and last for 1 month in some year (Zhou and Yu, 2007), and pose serious impact on costal marine ecosystem. The bloom of this species is result of a complex ecological and oceanographic process, which is affected by various environmental factors, such as nutrient availability (Ou et al., 2010; Zhang et al., 2015), temperature and salinity (Xu et al., 2010). However, the regulation of population growth and biomass accumulation of this species is still poorly understood. It is very challenging to monitor the development of the bloom in the natural marine environment largely due to lack of proper biomarkers to indicate growth status of the population. As the first step to address the gap of knowledge, in this study, we isolated G1/S cyclin, cellulose synthase and cellulase gene in *P. donghaiense*, and characterized their expression patterns in synchronized cultures due to dinoflagellates are in general known to be synchronized to the diel cycle in the field (Van Dolah and Leighfield, 1999). The expression dynamics of these genes indicated a tight association of the G1/S cyclin with the G1/S transition, cellulose synthase with the growth of newly divided cells, and cellulose with cell division. Our results suggest that these genes are promising candidates of cell cycle biomarkers for assessing growth status of *P. donghaiense*.

## Materials and Methods

### Algal Culture and Sample Collection for Gene Isolation

*Prorocentrum donghaiense* culture was provided by the Center for Collections of Marine Algae, College of Ocean and Earth Sciences, Xiamen University (Shi et al., 2015). The culture was grown in autoclaved L1 seawater medium (without silicate) at 20 ± 1°C, under a 14 h/10 h light/dark cycle with a photon flux of 100 μE⋅m^-2^⋅s^-1^. A cocktail of antibiotics (Lin et al., 2015) was provided to minimize bacterial presence. The growth rate of the culture was monitored using a Sedgwick-Rafter counting chamber under a microscope. Exponential phased cells (∼10^7^ cells per sample) were harvested by centrifugation at 3000 × *g* under 20°C for 10 min. For RNA isolation, the cell pellets were resuspended in 1 ml TRIzol Reagent (Invitrogen, Carlsbad, CA, United States) thoroughly by vortex and stored at -80°C for subsequent RNA extraction. For DNA extraction, cell pellets were fixed with 400 μL DNA lysis buffer (contains 10 mM Tris pH 8.0; 100 mM EDTA pH 8.0; 0.5% SDS with 200 μg/ml proteinase K) and stored at -20°C for DNA isolation.

### Diel Sample Collection for Gene Expression and Flow Cytometric Analysis

Culture was first synchronized as previously reported (Shi et al., 2013). The synchronized culture was then transferred into 7.5-L L1 medium in triplicate. The culture condition was the same as described above. When the culture entered early exponential phase (3 days after transfer), a sample was taken every 2 h for a 24 h period. At each time point, 400 and 100 ml samples were harvested as described above for RNA extraction and flow cytometric analysis, respectively. Cell pellets for RNA extraction were suspended in 1 ml TRIzol Reagent and stored at -80°C as described above. Flow cytometry cell pellets were fixed by 1 ml 70% ethanol solution and stored at -20°C until analysis.

### Flow Cytometric Analysis of the Cell Cycle

Sample processing, DNA staining, and flow cytometric cell cycle analysis were carried out as previously reported (Li et al., 2015). Briefly, the ethanol fixed samples were washed with 1× PBS (phosphate buffered saline, pH = 7.4), and resuspended in 1 mL ice cold 100% methanol and stored at 4°C for 12 h to extract pigments whose auto-fluorescence could otherwise interfere with DNA fluorescence measurement. The cells were washed again and resuspended with 0.5 ml propidium iodide (PI; Sigma, St. Louis, MO, United States) staining solution, and incubated in the dark to digest RNA and stain DNA. Cell cycle analysis of the PI-stained cells were carried out on Cell Lab Quanta SC flow cytometer (Beckman Coulter Inc., United States) with 488 nm excitation and 576 nm emission, and data were collected from 20,000 randomly encountered cells for each sample. The cell cycle profile in each sample was analyzed using ModfitLT software to generate percentage of each cell cycle phase in the samples with debris population were removed.

### DNA and RNA Extraction and cDNA Synthesis

DNA lysis buffer fixed sample was incubated for 3 days at 55°C, followed by bead beating (Yuan et al., 2015). DNA extraction was then carried out using a CTAB protocol combined with Zymo DNA Clean & Concentrator kit (Zymo Research Corp., Orange, CA, United States) as previously reported (Zhang et al., 2005).

Total RNA was extracted using TRI-Reagent (Molecular Research Center, Inc., Cincinnati, OH, United States) coupled with Qiagen RNeasy Mini kit (Qiagen) following previously reported protocol (Lin et al., 2010). To eliminate potential genomic DNA contamination, the RNA preparations were treated with RQ1 DNase (Promega) according to the manufacturer’s protocol and further purified using Qiagen RNeasy Mini kit. RNA concentrations were measured using NanoDrop ND-2000 Spectrophotometer (Thermo Scientific, Wilmington, DE, United States), and the quality was assessed using the absorbance ratios of 260/280 nm and 260/230 nm.

For qPCR to quantify specific gene expression, first-strand cDNA was synthesized from 300 ng total RNA for each sample using M-MLV reverse transcriptase (Promega, Madison, WI, United States) and 100 ng oligo-(dT)16 primers. In the experiment to isolate gene cDNA sequences, GeneRacer oligo-dT (Invitrogen, Carlsbad, CA, United States) and modified random N9 primers were used separately to construct cDNA libraries (named Racer3-cDNA and N9-cDNA, respectively).

### Gene Isolation and Sequence Analysis

Gene specific primers (**Table 1**) were designed based on annotated partial cDNA sequences of RNA-Seq dataset (unpublished data). PCR with the synthesized cDNA as the template and the primers designed here was carried out to amplify G1/S cyclin, cellulose synthase and cellulose coding genes. The amplicons were purified and sequenced. To obtain full-length cDNAs of these genes, specific primers were designed from these sequences to pair with Racer3 and DinoSL to isolate 3-end and 5-end of gene, respectively (the NCBI accession number of the three genes are KY926427–KY926429). Racer3-cDNA and N9-cDNA libraries were used in PCR separately as templates in PCR. The amplicons were purified and sequenced. These sequences and those of the fragments obtained initially were assembled to generate full-length gene sequences.

**Table 1:** Primers used in this study.

Primer name	Sequences (5′–3′)	Application
Pd_CYC_F	TGGCCCGCGTGTTGAGCCACTTGT	G1/S cyclin gene PCR (forward)
Pd_CYC_R	CCTCGCAGCAATGAGCACGTGGT	G1/S cyclin gene PCR (reverse)
Pd_CYC_qF	AGCAACGCGTGCCTGATCGCGA	G1/S cyclin gene qPCR (forward)
Pd_CYC_qR	GAGCATTCAGCTCCTGCAACTC	G1/S cyclin gene qPCR (reverse)
Pd_CLA_F	CATCTGGGAGGCGAACTCTATGG	Cellulase gene PCR (forward)
Pd_CLA_R	CGGTGTACGTGGACCCGATCTCC	Cellulase gene PCR (reverse)
Pd_CLA_qF	GTGGAATGACACTGGTCCTGTC	Cellulase gene qPCR (forward)
Pd_CLA_qR	TACGTCGTAGACGCGTCCGGGTA	Cellulase gene qPCR (reverse)
Pd_CESA_F	TGCCCGAGAACGTAGCGGCTTCGA	Cellulose synthase gene PCR (forward)
Pd_CESA_qF	GATTATGCTGTTCGTGGTGGCGA	Cellulose synthase gene qPCR (forward)
Pd_CESA_qR	CGTATGTAAGCAGCATGTAGC	Cellulose synthase gene qPCR (reverse)
Dino-SL	TCCGTAGCCATTTTGGCTCAAG	mRNA 5′ end cDNA synthesis and PCR (forward)
GeneRacer3	GCTGTCAACGATACGCTACGTAACG	mRNA 3′ end cDNA synthesis and PCR (reverse)
GeneRacer oligo-dT	GCTGTCAACGATACGCTACGTAACGGCATGACAGTG(T)24	cDNA library construct

To tentatively predict the subcellular localization of the G1/S cyclin, cellulose synthase, and cellulose, the sequences obtained in this study as well those found in RNA-Seq dataset (unpublished data) were first analyzed by BLAST against GenBank nr database. The protein cording region of each gene was predicted by ORF Finder (Rombel et al., 2002). The obtained protein sequence was analyzed by InterProScan (Zdobnov and Apweiler, 2001) to predict potential functional domains. SignalP^1^ was then used to predict N-terminal signal peptide.

### Diel Gene Expression Analysis Using Reverse Transcription Quantitative PCR (RT-qPCR)

The expression levels of the three genes in diel samples were determined using reverse transcription quantitative PCR (RT-qPCR). A cDNA fragment was amplified for each gene using primer designed for qPCR (**Table 1**), and the amplicons were then cloned and PCR-amplified again to yield standards (Hou et al., 2010). These standard DNAs were serially diluted by 10-folds to be included in the qPCR runs (Shi et al., 2013). qPCR was performed in 96-well plates on a CFX96 Real-time PCR System (BioRad, United States) with iQTM SYBR^®^ Green Supermix (BioRad, United States). For both the 10-fold serial dilution standard and the experimental cDNA, qPCR was run in triplicate (for technical replicates) for each of the biological triplicate. GAPDH (Glyceraldehyde 3-phosphate dehydrogenase) was included in the qPCR as a reference gene for normalization of target gene expression levels (Shi et al., 2015). The transcript abundances of the target genes and the reference gene were analyzed using CFX software (Bio-Rad, Hercules, CA, United States).

### Quantification of Gene Copy Number Using qPCR and Analysis of Correlation with Maximal Expression Level

There is a growing interest in correlating variation in gene copy number with expression and function of the gene for various organisms (Bachvaroff and Place, 2008). To explore the possibility that the different gene expression levels exhibited by the genes studied here are determined by their respective gene copy numbers, specific gene copy number in the genome was determined using qPCR as described for gene diel expression. The standard and reaction system used were the same as described above except that the template was genomic DNA (2, 10, and 50 ng was used in separate reactions for each gene). Results from these three quantities of the template were normalized to per ng genomic DNA, which were then converted to copy number per cells based on genome size of *P. donghaiense*. The data on the genomic copy numbers and expression (transcriptional) data on these genes were analyzed to seek their correlations. To broaden the correlation analysis, we also included previously reported gene copy number and maximal expression data of RuBisCo and rhodopsin (Shi et al., 2013, 2015).

### Phylogenetic Analysis

Phylogenetic trees were constructed to determine the affiliation of the target genes with previously characterized genes based on their amino acid sequences. NCBI nr and MMETSP database (Keeling et al., 2014) were used to obtain reference protein sequences by BLAST search. Alignment of these sequences was carried out using ClustalW of MEGA5 (Tamura et al., 2011). Poorly aligned segments were removed from phylogenetic tree analysis. Phylogenetic tree was inferred using the Maximum Likelihood (ML) method in MEGA5, with 500 resampling in bootstrapping. JTT amino acid substitution method was selected in MEGA software package.

## Results

### Cell Growth and Cell Cycle of *P. donghaiense*

**FIGURE 1:**
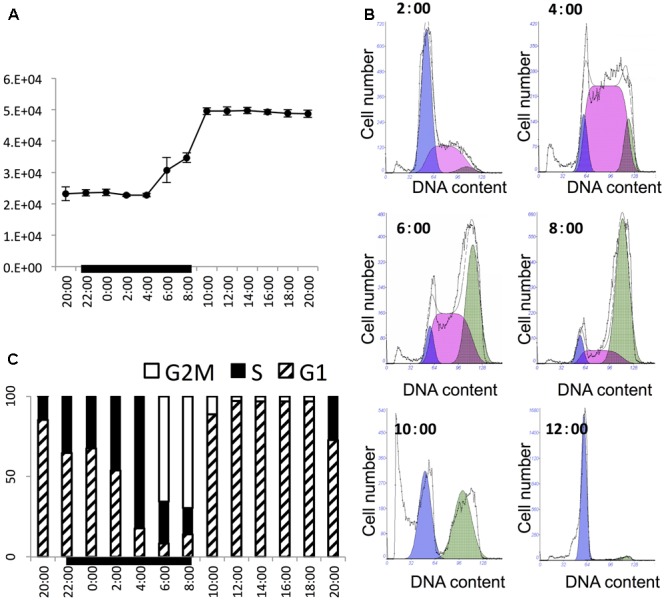
Cell growth pattern and cell cycle of *P. donghaiense* in the diel cycle. **(A)** Diel growth pattern of *P. donghaiense* in the synchronized culture. **(B)** Flow cytometric profile of key time points of cell division. The colored area indicate fitted cell cycle phase, purple appears to be for G1, pink for S and green for G2M phase. **(C)** Diel cell cycle profiles of *P. donghaiense* in the sampling period. The bar graph was derived from the flow cytometric DNA content.

The culture grew exponentially in the 24-h sampling period. The initial cell concentration was about 21,000 cells mL^-1^ and the final cell density was about 48,000 cells mL^-1^, with 2.11-fold increase within 1 day (**Figure 1A**). The cell density stayed largely unchanged in the first five sampling time points (from 2 h before till 6 h after light switch-off), then ramped up sharply to reach a high level in 6 h (2 h after light switch-on). Thereafter, the cell density was maintained relatively stable until next cycle of cell proliferation. This stepwise growth pattern indicated that the culture was highly synchronized and cell division occurred between 4 h before and 2 h after the onset of the light period in the 14 h/10 h light/dark cycle (4:00–8:00). This was verified by the change in the flow cytometric DNA profiles at several time points in this period (**Figure 1B**), showing the rapid increase of the S phase at 4:00 succeeded by that of the G2M phase from 6:00 to 8:00. Further detailed cell cycle analysis (**Figure 1C**) indicated that the culture started to enter the S phase as early as 2 h before light switch-off (20:00). The percentage of S-phase cells increased steadily until the 6th hour of the dark period (4:00) to around 81%. After that, cells entered into the G2M phase, with S-phase cells declining and G2M phase reaching the peak at the end of the dark period (8:00). Then the culture entered into G1 phase rapidly and stayed in G1 for the entire light period, ready for the next cycle (**Figure 1C**).

### Gene Sequence and Phylogenetic Analysis

Gene fragments of G1/S cyclin, cellulose synthase and cellulase were generated from an RNA-Seq of *P. donghaiense* (unpublished data). The full-length cDNA sequences of G1/S cyclin and cellulose were isolated using DinoSL- (for 5′-end) and Racer3- (for 3′-end) based PCR, which were 815 and 2570 bp long, respectively. Our attempt to obtain the full long cDNA of cellulose synthase failed due to low PCR efficiency. However, from our RNA-Seq transcript assembly, we identified a cDNA sequence encoding 1174 amino acid (aa) residues.

**FIGURE 2:**
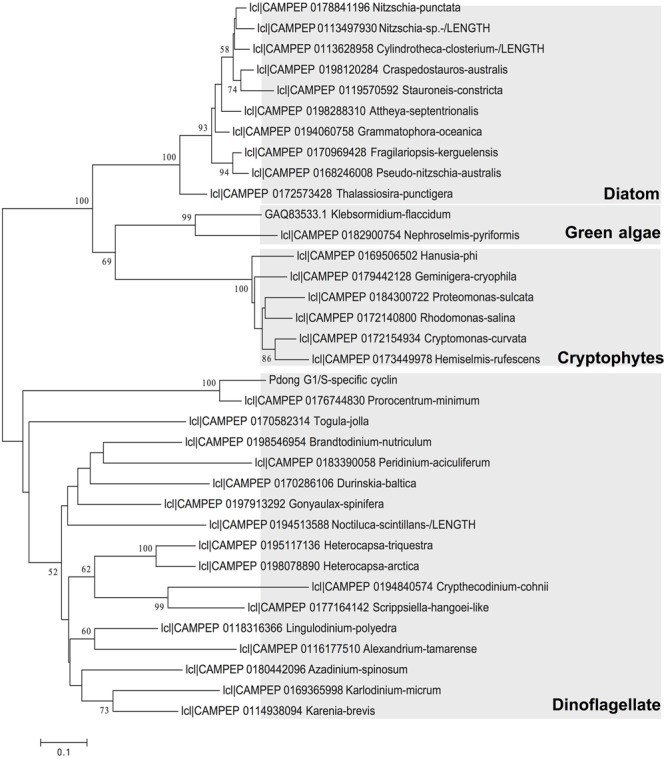
Phylogenetic relationship of *P. donghaiense* G1/S cyclin gene with this gene in other species based on amino acid sequences. Tree topology shown is from Maximum Likelihood (ML) analysis. Values shown at nodes are NJ bootstrap values of ML analyses (only values >50% are shown).

ORF Finder indicated that the G1/S cyclin cDNA encoded a 202-aa protein, with a predicted molecular weight of 22.06 kDa. BLAST against NCBI nr database analysis returned *Perkinsus marinus* G1/S-specific cyclin PCL5 as the top hit, with 45% identity. Gene structure predication showed a conserved cyclin box domain (**Supplementary Figure S1**) located at residues 84 to 168 of the deduced protein sequence. This cyclin box region was conserved across dinoflagellate sequence reported in MMETSP (Keeling et al., 2014). Phylogenetic analysis of amino acid sequences showed that the cyclin we isolated was affiliated with counterparts in dinoflagellates, and in general homologs from each phylum of phytoplankton formed a monophyletic clade. In the clade of dinoflagellates the sequence of *P. donghaiense* formed a strongly supported subclade with *P. minimum* (**Figure 2**). The branches of dinoflagellate species are longer than other phytoplankton species, indicating a high evolutionary rate of this gene in this phylum of microalgae.

The 674-aa *P. donghaiense* cellulase cDNA was predicted to code for a protein of approximately 71.25 kDa. InterProScan search and structure predication indicated that this protein belonged to Glycosyl hydrolase family 7. The deduced domain region showed 55% similarity to the corresponding domain in dinoflagellate *Lingulodinium polyedrum* (ADG63074) cellulase gene, which was the top hit of BLAST search in NCBI nr database. A signal peptide was identified in the N terminus using SignalP 4.1^2^, indicating similar structure to cellulase in *L. polyedrum* (Kwok and Wong, 2010). The most likely cleavage site of this enzyme was between residues Ala–Gln at positions 16 and 17 (**Supplementary Figure S2**).

Using 3′-RACE, we successfully isolated the C-terminus of cellulose synthase gene using PCR. The C-terminal sequence was 100% identical to the transcript of this gene generated by RNA-Seq, suggesting high quality of RNA-Seq assembly. Sequence analysis of the full-length assembly indicated that this cDNA had an open reading frame of 3522 bp, which encoded a protein of 1174 amino acids with a predicted molecular mass of approximately 129.22 kDa. By InterProScan search and structure predication, we found five transmembrane domains in the C-terminal region of the protein and two transmembrane domains in the N-terminal region. This specific structure is common in cellulose synthase superfamily (Richmond and Somerville, 2000). The prediction also indicated that this protein would be localized to the plasma membrane, since C-terminus and N-terminus of this protein contains cytoplasmic and non-cytoplasmic domain, respectively (Delmer and Amor, 1995), similar to the homolog in the land plant *Arabidopsis* (Richmond and Somerville, 2000).

### Diel Expression of G1/S *Cyclin, Cellulose Synthase, Cellulase*

**FIGURE 3:**
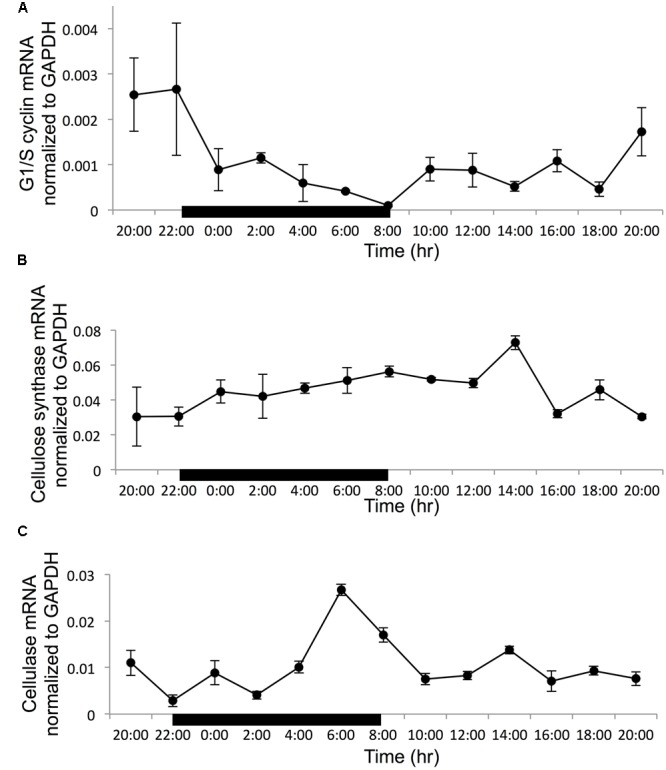
Gene transcription dynamics of specific genes normalized to reference gene GAPDH in a cell cycle (black bar indicate dark period, other time point is light period). **(A)** G1/S cyclin; **(B)** cellulose synthase; **(C)** cellulose. Error bars indicate ± standard deviation.

Normalized to the reference gene GAPDH, all the three target genes showed a clear diel rhythm in the 24-h sampling period (**Figure 3**). For the G1/S cyclin, the transcript abundance decreased steadily in the dark period to reach the minimum at the time point of dark/light transition (8:00), when the cell population was dominantly in G2M. Then it remained low until dusk when it started to increase to reach the maximum at the light/dark transition when S phase started to increase remarkably (**Figure 3A**). Overall, the expression of G1/S cyclin showed a 26-fold variation throughout the whole sampling period. The gene expression difference between 22:00 and 8:00 h has a 26-fold difference (with the ratio compared to the reference at 22:00 being 0.0026 and the ratio compared to the reference at 8:00 is 0.0001). For cellulose synthase encoding gene, the transcript abundance was relatively stable in the whole diel cycle, although there was a small trend of increase throughout the dark period and the first 6 h of the light period, with an apparent peak at the 6th hour of the light period (G1 phase) (**Figure 3B**). The transcript abundance showed a 2.4-fold variation in the entire sampling period. For the cellulase gene, the transcript abundance increased rapidly toward the end of the dark period reaching its peak level 2 h before the dark/light transition (G2M dominant), and decreased thereafter (**Figure 3C**). The expression of this gene showed a 9.3-fold dynamic range throughout the sampling period.

### The Transcriptional Levels of G1/S *Cyclin, Cellulose Synthase* and *Cellulase* and Correlation with Their Genomic Copy Number in *P. donghaiense* Genome

In some dinoflagellates, the specific gene expression level can be estimated from their genomic copy number based on the correlation established between them (Lee et al., 2014). To explore if this applies to the three genes we studied here, we determined copy numbers for these three genes. A highly linear and high-efficiency standard curve was generated for each specific gene (**Supplementary Figure S3**). Based on the standard curve, qPCR for templates of 2, 10 and 50 ng *P. donghaiense* genomic DNA were used to estimate gene copy number of each of the three genes. The result indicated that there were 11206 ± 780, 2528 ± 611 and 1579 ± 175 copies for G1/S cyclin, cellulose synthesis and cellulase genes, respectively, in 1 ng genomic DNA on average. According to previous reported genome size of *P. donghaiense* (Shi et al., 2013; Yuan et al., 2015), 1 ng genomic DNA is equivalent to 148.6 ± 25.2 cells. These values yielded 75.4 ± 5.2, 17.9 ± 4.1 and 10.6 ± 1.2 copies per cell for the G1/S cyclin, cellulose synthase and cellulase, respectively.

**FIGURE 4:**
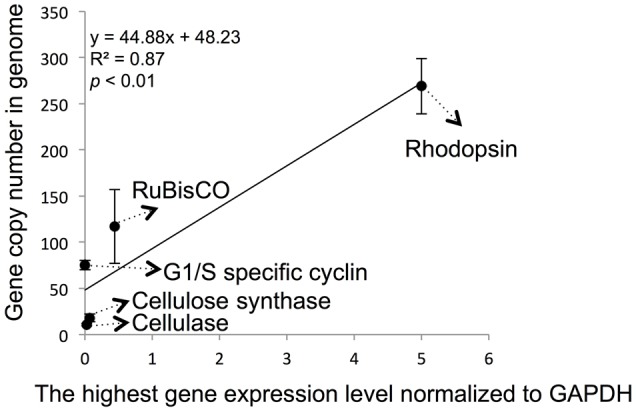
Correlation between specific gene maximal expression level and genomic gene copy number in *P. donghaiense*. Five genes, including RuBisCo, rhodopsin, cellulose synthase, cellulase and G1/S cyclin, were used in the correlation analysis. Relative expression level and gene copy number were estimated by qPCR. The highest expression level of each gene in diel samples was selected to do analysis. Error bars indicate ± standard deviation of gene copy number in genome.

We also attempted to correlate the genomic copy numbers of the genes with their maximal transcriptional levels, and found that the gene copy numbers for cellulose synthase and cellulose genes were similar, their maximal expression levels determined by RT-qPCR were also close (**Figure 4**). However, even though G1/S cyclin showed a markedly higher genomic copy number, its maximal expression throughout the cell cycle was not significantly higher (*p* > 0.05, *t*-test). When the previously reported data on RuBisCO and rhodopsin in *P. donghaiense* (Shi et al., 2013, 2015) was added to the analysis, a positive linear correlation was observed (**Figure 4**; *R*^2^ = 0.87 and *p* < 0.01).

## Discussion

Like many other species of dinoflagellates, *P. donghaiense* is a harmful bloom-forming species. While it is well recognized that cell proliferation is a major cause of the blooms (Anderson et al., 2012), how cell proliferation is regulated at the molecular level is still poorly understood. Consequently molecular markers that would be very useful for monitoring bloom dynamics are lacking. In this study, we obtained gene sequences and characterized expression dynamics of the G1/S cyclin, cellulose synthase and cellulase because they are potentially associated with cell division cycle. Flow cytometric analysis of the cell cycle coupled with RT-qPCR measurement of gene expression levels allowed us to document the diel gene expression dynamic of these genes throughout the cell cycle. Similar studies have been limited to proliferating cell nuclear antigen in *Pfiesteria piscicida* (Zhang et al., 2006) and mitotic cyclin or cyclin B in *Karenia brevis* (Barbier et al., 2003), *L. polyedrum* (Bertomeu and Morse, 2004) and *Alexandrium fundyense* (Zhuang et al., 2013). More information on regulators of different time points in the cell cycle would contribute toward a better understanding of regulation of the cell cycle and population growth in dinoflagellates and offer potentially useful molecular markers.

### G1/S Cyclin in Association with G1-to-S Transition

G1/S transition is the point where the cell checks whether it has enough raw materials, which were synthesized based on available nutrient in the surroundings, to fully replicate its DNA. Thus G1/S transition is described to be a rate-limiting step for cell proliferation (Edgar, 1994) and is also known as the restriction point in the cell cycle. Therefore, the G1/S specific cyclin can potentially be used as a growth rate marker for harmful algal bloom research. This is the first study that reports isolation and transcriptional regulation of G1/S specific cyclin in a dinoflagellate. The BLAST search in NCBI nr database showed that the top hit was G1/S-specific cyclin in *P. marinus*, a phylogenetically close relative of dinoflagellate (Lin, 2011). Phylogenetic analysis showed that this cyclin in dinoflagellates formed a monophyletic clade but with longer branches than that in other clades. This indicates that the G1/S cyclin is common in dinoflagellates and the perkinsids, but with higher sequence variations in comparison to other phyla of algae. Our diel expression data showed that the G1/S specific cyclin gene expression level rose in late G1 and fell in early S phase. This expression profile is consistent with G1/S cyclin in yeast (Lew and Reed, 1992), and agrees with the expected role of this protein in mediating the G1-to-S phase transition, which induces the initial processes of DNA replication for cell proliferation. In contrast, the mitotic cyclin or cyclin B previously reported in other dinoflagellates has a peak transcript abundance in the G2M phase (Zhuang et al., 2013), in accordance with its expected function in regulating the G2-to-M progression. The sequence structure, phylogenetic affiliation, and expression profile unraveled in the present study indicate that the gene we identified is a typical G1/S specific cyclin, likely mediating G1 to S phase transition in *P. donghaiense*.

### Cellulose Synthase in G1 and Cellulase in G2/M

Dinoflagellates usually possess internal cell wall system as in euglenids and cryptomonads (Morrill and Loeblich, 1983). The main constituent of cell wall is the amphiesma filled with cellulose fibrils. The cellulosic materials are synthesized by cellulose synthase, an enzyme localized in the plasma membrane (Giddings et al., 1980). Bioinformatics analysis of *P. donghaiense* cellulose synthase gene indicated that this protein possesses multiple transmembrane domains, which is barred in plasma membrane as previously reported (Delmer and Amor, 1995). The C-terminus of the peptide would hang out in cytoplasm, and the N-terminus would be embedded in the cell wall.

In certain bacteria, cellulose synthase is directly regulated by cyclic-di-GMP (Cotter and Stibitz, 2007), a universal bacterial second messenger that also regulates the cell cycle (Römling et al., 2013). Cyclic-di-GMP usually acts as a cyclin-like molecule to control cell cycle progression in bacteria (Lori et al., 2015). Such an equivalent regulatory system has not been described in eukaryotes. In dinoflagellate and other eukaryotes, cell cycle is regulated by cyclins and CDKs. Only limited information has been reported about the correlations among cellulose synthase, cell cycle and cyclin. In *L. polyedrum*, flow cytometric analyses indicated that cellulose accumulation was a stepwise process in the cell cycle, with the highest content occurring at G1 phase (Kwok and Wong, 2003). In our result, the transcript abundance of cellulose synthase was kept at a relatively high level throughout the diel cycle (cell cycle) with a small peak at the middle of the G1 phase, probably indicating a need for constantly active synthesis of cellulose while a higher demand during the G1 phase. Based on these results, it is apparent that cellulose synthase gene in *P. donghaiense* is G1 phase associated and the highest cellulose fibril synthesis rate occurs in the G1 phase.

The function of cellulase is to hydrolyze oligosaccharides and/or polysaccharides into glucose (Sukumaran et al., 2005). This gene in *P. donghaiense* belongs to the glycoside hydrolase family 7. It functionally pairs with cellulose synthase and is believed to occur even in athecate species to provide an important precondition for the acquisition of cellulosic thecal plates (Janouškovec et al., 2016). Exoglucanases and cellobiohydrolases are the two main activated enzymes in the glycoside hydrolase family 7. Recently reported dinoflagellate cellulases are all exoglucanases (Kwok and Wong, 2010), including that in *P. donghaiense*.

The expression profile of cellulase has been studied extensively in fungi and plants. Cellulase gene expression is tightly controlled at the transcriptional level in fungal (Coradetti et al., 2012). In *Polyporus arcularius*, the transcript abundance of cellulase is found to be consistently at a very low level, but can be induced by endoglucanase production (Ohnishi et al., 2007). In higher plants, cell growth is promoted by cellulase overexpression (Park et al., 2003) and prevented by suppression of this gene (Hayashi et al., 2005). Cellulase has also been reported to play a role in cell elongation (Ohmiya et al., 2003). In *L. polyedrum*, cellulase is mainly located in cell wall and its activity increase significantly in G2/M phase (Kwok and Wong, 2010), which coincides with cellulose drop in this period of cell cycle (Kwok and Wong, 2003). Cell cycle inhibition experiments on *L. polyedrum* indicate that cellulose synthesis and degradation are tightly regulated in the cell cycle (Kwok and Wong, 2003, 2010). In our result, the highest expression level of cellulase gene was detected in the G2M phase. In the two sampling time points where G2M phase cells were predominant in the culture, cellulase transcript abundance was 3.3- and 2.1-fold higher, respectively, than the average for the rest of the diel cycle when the cell population was in the other cell cycle phases. This is consistent with the significant increase in cellulase abundance in G2M phase in *L. polyedrum* (Kwok and Wong, 2010). It has been illustrated that cellulase plays a role upstream of the cell size-sensing mechanism and coordinates cell wall degradation with cell cycle progression (Kwok and Wong, 2010). Since the largest cell size occurs in the G2M phase in dinoflagellate (Zhuang et al., 2013; Li et al., 2016), it is possible that the expression of *P. donghaiense* cellulase was regulated by cell cycle also in a cell size-dependent manner. Considering the results of cellulose synthase and cellulose gene expression together, we postulate that cellulose synthesis takes place throughout the cell cycle, while its breakdown mainly occurs at the G2M phase.

### Gene Expression Level in Correlation with Genomic Copy Number

Based on the expression profile of the three genes we tested, their expression levels showed large variation. For instance, the highest expression level of G1/S cyclin, cellulose synthase and cellulase in cell cycle dynamic are 0.0027, 0.0728, and 0.0267, respectively. The expression level of cellulose synthase is 26-fold higher than that in G1/S cyclin. It is of interest to know if gene copy variation can explain the differences in the expression level of these genes because gene copy number has increasingly been studied to understand if it influences gene expression level and function (Lee et al., 2014). In dinoflagellates high expression level of specific gene suggests high demand of its encoded protein, which usually shows high genomic copy number (Zhang and Lin, 2003; Zhang et al., 2006). Genome sequence survey of the larger-genome species *Heterocapsa triquetra* indicted that different functional genes usually occur in great and variable copies (McEwan et al., 2008). We found cellulose synthase and cellulase genes had similar copy numbers in the *P. donghaiense* genome and have similar maximal expression levels. Yet although the G1/S cyclin gene had a markedly higher copy number, its maximal expression level we detected was not significantly higher. This renders it unclear whether the dramatically higher copy number of the G1/S cyclin translates to higher abundance of the encoded protein in comparison to cellulose synthase and cellulase genes. When data on the carbon fixing enzyme Rubisco and the putative energy converting proton pump rhodopsin are included, there seem to be a positive correlation. This is agreeable with a correlation previously found in the dinoflagellate *Oxyrrhis marina* (Lee et al., 2014). Whether such correlation is a common feature of dinoflagellates and whether this can help predict expression levels of genes through determining genomic copies warrant further investigation in the future.

## Author Contributions

Conceived and designed the experiments: XS, SL, and MM. Performed the experiments: XS and MM. Analyzed the data: XS and SL. Wrote the paper: XS and SL.

## Conflict of Interest Statement

The authors declare that the research was conducted in the absence of any commercial or financial relationships that could be construed as a potential conflict of interest. The reviewer DW declared a shared affiliation, though no other collaboration, with the authors to the handling Editor, who ensured that the process nevertheless met the standards of a fair and objective review.

## Footnotes

1 www.cbs.dtu.dk/services/SignalP/

2 www.cbs.dtu.dk/services/SignalP/

## Supplementary Material

FIGURE S1 Sequence alignment of cyclin box region of G1/S cyclin in dinoflagellate.

FIGURE S2 N-terminal of *P. donghaiense* cellulase and its signal peptide cleavage site.

FIGURE S3 Copy number of gene cellulase **(A)**, cellulose synthase **(B)** and G1/S cyclin **(C)** in *P. donghaiense* genome were estimated using qPCR. Standard curve (open circle and the line) was constructed using decadal dilution series of respective cDNA, from 10^7^ to 10^3^ copies, each in triplicate. Three different amounts of *P. donghaiense* genomic DNA (DNA equivalent to 2, 10, and 50 ng), each in triplicate, were used as unknown samples, which gave estimate of 75.4 ± 0.52, 17.9 ± 0.41 and 10.6 ± 0.12 copies of G1/S cyclin, cellulose synthase and cellulase, respectively, in one cell.
