# Supplementary figures and images for: Cell Cycle-Dependent Expression Dynamics of G1/S Specific Cyclin, Cellulose Synthase and Cellulase in the Dinoflagellate Prorocentrum donghaiense
Source: Front Microbiol. 2017 Jun 20;8:1118. doi: 10.3389/fmicb.2017.01118 (PMC5476699; doi:10.3389/fmicb.2017.01118)

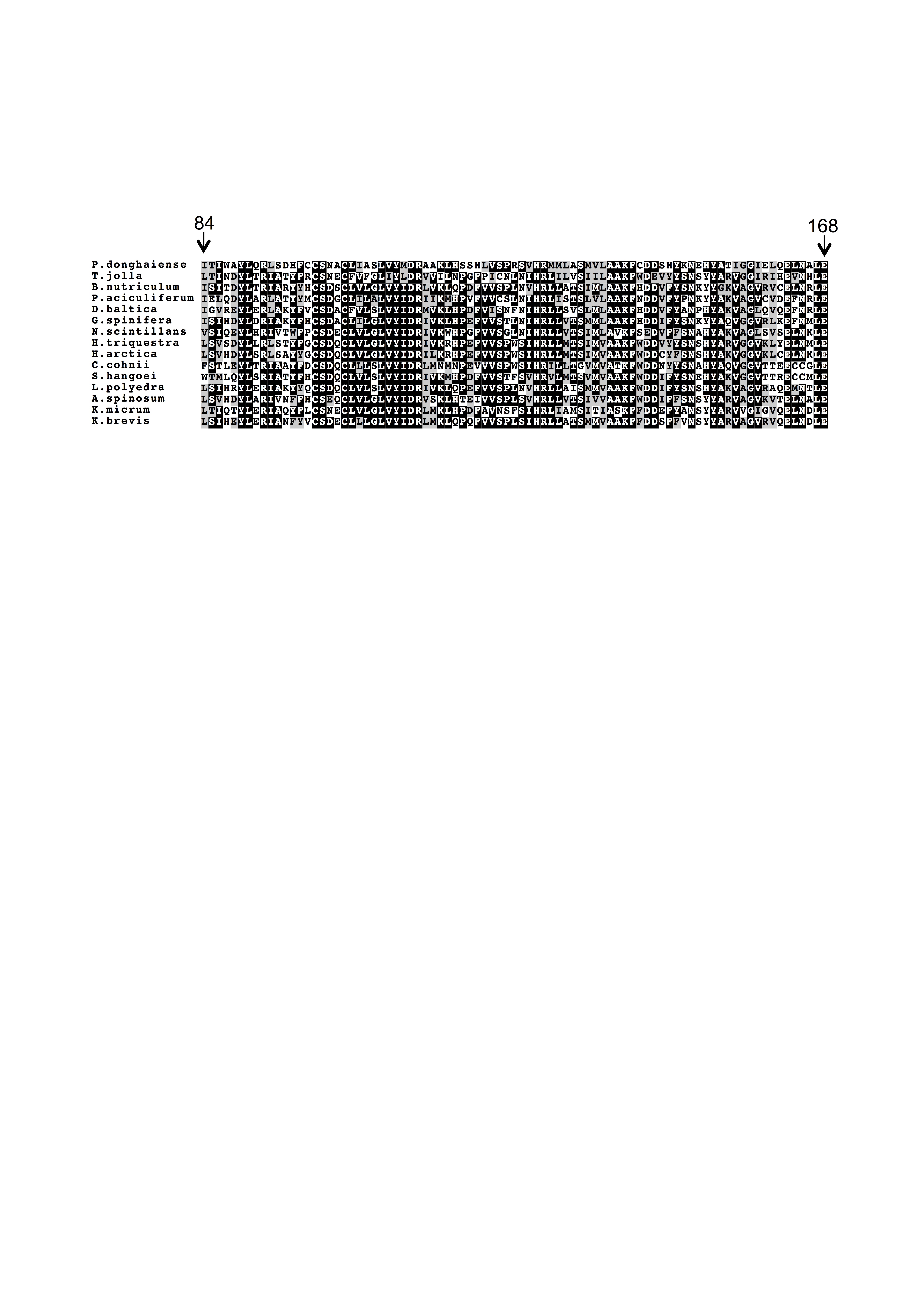

Supplement: FIGURE S1 — Sequence alignment of cyclin box region of G1/S cyclin in dinoflagellate. [file Image_1.JPEG]

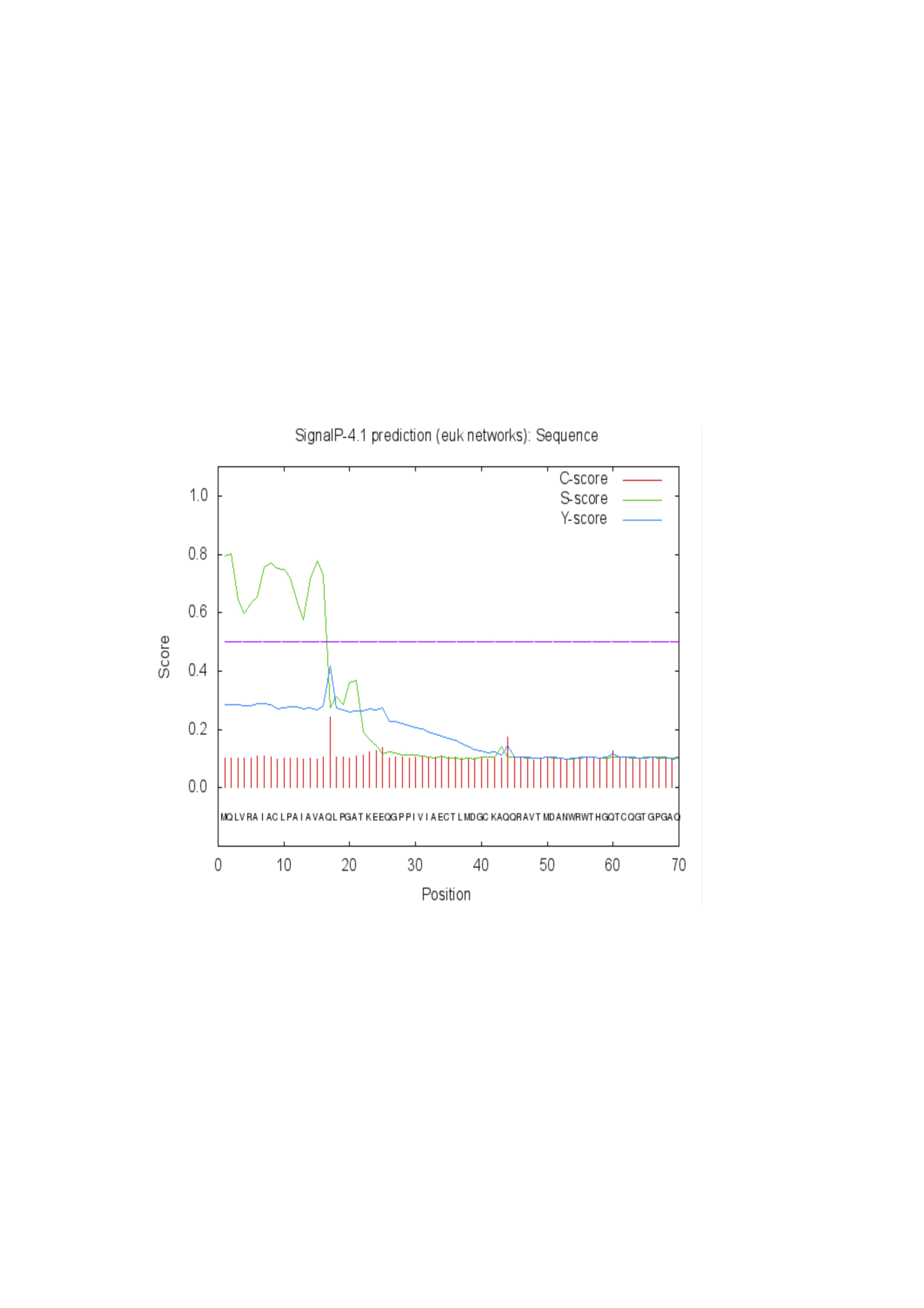

Supplement: FIGURE S2 — N-terminal of P. donghaiense cellulase and its signal peptide cleavage site. [file Image_2.JPEG]

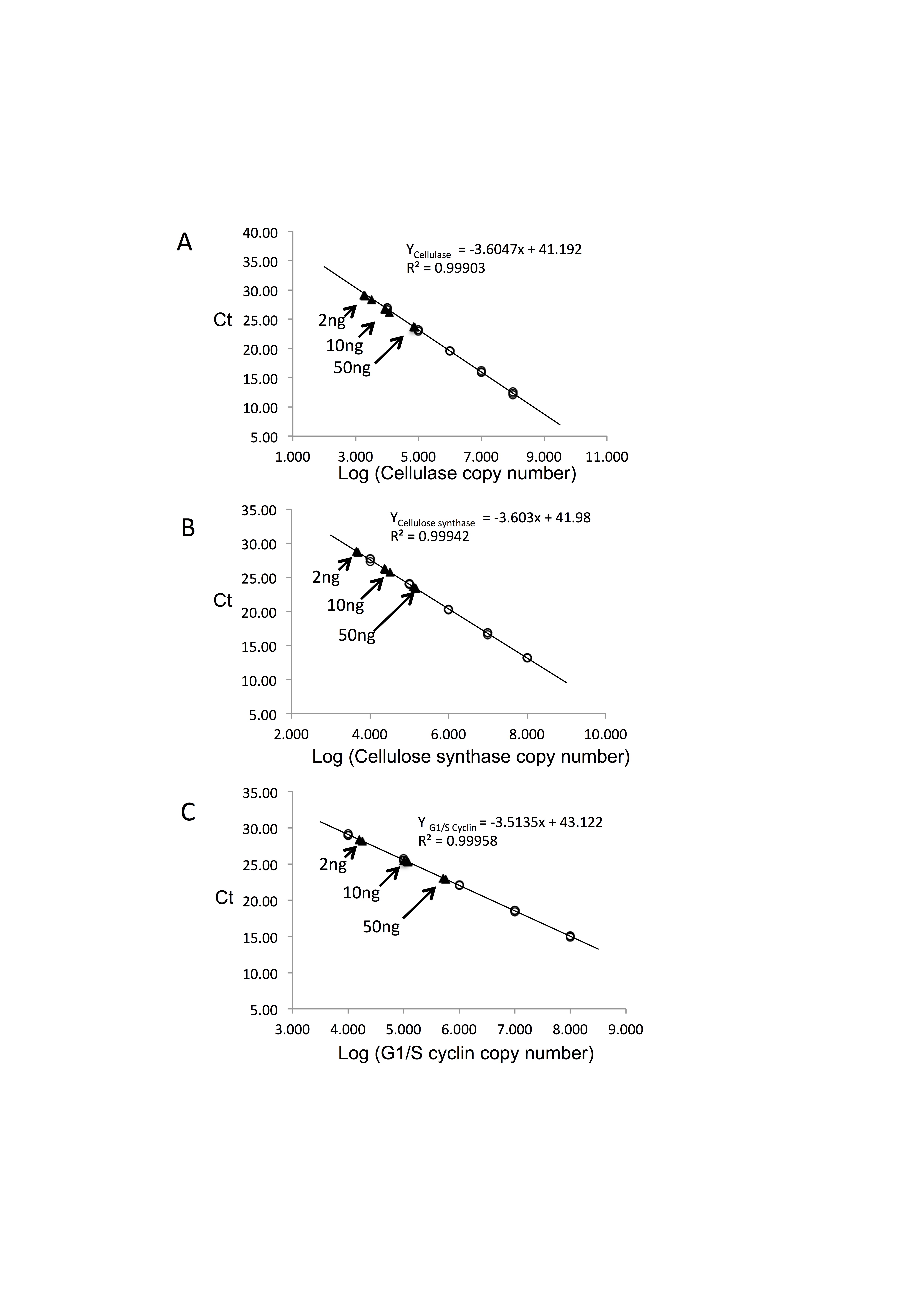

Supplement: FIGURE S3 — Copy number of gene cellulase (A), cellulose synthase (B) and G1/S cyclin (C) in P. donghaiense genome were estimated using qPCR. Standard curve (open circle and the line) was constructed using decadal dilution series of respective cDNA, from 107 to 103 copies, each in triplicate. Three different amounts of P. donghaiense genomic DNA (DNA equivalent to 2, 10, and 50 ng), each in triplicate, were used as unknown samples, which gave estimate of 75.4 ± 0.52, 17.9 ± 0.41 and 10.6 ± 0.12 copies of G1/S cyclin, cellulose synthase and cellulase, respectively, in one cell. [file Image_3.JPEG]
